# Integrative molecular profiling indicates a central role of transitory starch breakdown in establishing a stable C/N homeostasis during cold acclimation in two natural accessions of *Arabidopsis thaliana*

**DOI:** 10.1186/s12870-015-0668-1

**Published:** 2015-12-01

**Authors:** Matthias Nagler, Ella Nukarinen, Wolfram Weckwerth, Thomas Nägele

**Affiliations:** Department of Ecogenomics and Systems Biology, University of Vienna, Althanstr. 14, 1090 Vienna, Austria; Vienna Metabolomics Center (VIME), University of Vienna, Althanstr. 14, 1090 Vienna, Austria

**Keywords:** Cold acclimation, *Arabidopsis thaliana*, Natural variation, Starch metabolism, Amylases, Systems biology, Metabolomics, Proteomics, Phosphoproteomics, Growth regulation

## Abstract

**Background:**

The variation of growth and cold tolerance of two natural *Arabidopsis* accessions, Cvi (cold sensitive) and Rschew (cold tolerant), was analysed on a proteomic, phosphoproteomic and metabolomic level to derive characteristic information about genotypically distinct strategies of metabolic reprogramming and growth maintenance during cold acclimation.

**Results:**

Growth regulation before and after a cold acclimation period was monitored by recording fresh weight of leaf rosettes. Significant differences in the shoot fresh weight of Cvi and Rschew were detected both before and after acclimation to low temperature. During cold acclimation, starch levels were found to accumulate to a significantly higher level in Cvi compared to Rschew. Concomitantly, statistical analysis revealed a cold-induced decrease of beta-amylase 3 (BAM3; AT4G17090) in Cvi but not in Rschew. Further, only in Rschew we observed an increase of the protein level of the debranching enzyme isoamylase 3 (ISA3; AT4G09020). Additionally, the cold response of both accessions was observed to severely affect ribosomal complexes, but only Rschew showed a pronounced accumulation of carbon and nitrogen compounds. The abundance of the Cold Regulated (COR) protein COR78 (AT5G52310) as well as its phosphorylation was observed to be positively correlated with the acclimation state of both accessions. In addition, transcription factors being involved in growth and developmental regulation were found to characteristically separate the cold sensitive from the cold tolerant accession. Predicted protein-protein interaction networks (PPIN) of significantly changed proteins during cold acclimation allowed for a differentiation between both accessions. The PPIN revealed the central role of carbon/nitrogen allocation and ribosomal complex formation to establish a new cold-induced metabolic homeostasis as also observed on the level of the metabolome and proteome.

**Conclusion:**

Our results provide evidence for a comprehensive multi-functional molecular interaction network orchestrating growth regulation and cold acclimation in two natural accessions of *Arabidopsis thaliana*. The differential abundance of beta-amylase 3 and isoamylase 3 indicates a central role of transitory starch degradation in the coordination of growth regulation and the development of stress tolerance. Finally, our study indicates naturally occurring differential patterns of C/N balance and protein synthesis during cold acclimation.

**Electronic supplementary material:**

The online version of this article (doi:10.1186/s12870-015-0668-1) contains supplementary material, which is available to authorized users.

## Background

Plant growth together with stress tolerance and flowering traits are known to be orchestrated in a complex and interdependent molecular manner. Water supply, temperature and soil quality have been shown to be the most relevant abiotic factors which significantly affect these traits [1]. During the last decade, naturally occurring genetic and phenotypic variation of *Arabidopsis thaliana* has been shown to be a promising tool for studying the molecular architecture of such physiological traits. On the cellular level, abiotic stress affects the integrity of membrane systems, transport proteins, metabolic enzymes and signalling compounds, ultimately leading to disfunctions in cellular metabolism which directly impair plant growth and development. Previous studies have shown and discussed significant differences in naturally occurring stress tolerance, morphology, developmental programming and flowering of *Arabidopsis thaliana* [2–9].

Low temperature belongs to one of the most important abiotic factors limiting the geographic distribution of plants. In many temperate species, the exposure of plants to low but non-freezing temperatures initiates a process termed cold acclimation resulting in increased freezing tolerance [10]. The process of cold acclimation is a multigenic trait being characterized by a comprehensive reprogramming of the transcriptome, proteome and the metabolome, but also of enzyme activities and the composition of membranes [3, 11–17]. Particularly, reprogramming of primary metabolism plays a crucial role during cold acclimation leading to a changed photosynthetic activity and the accumulation of soluble sugars, amino acids and polyamines. Concentrations of the di- and trisaccharide sucrose and raffinose, respectively, have been shown to correlate well with winter hardiness in several plant species [18, 19]. Further, several roles for sugars in protecting cells from freezing injury have been proposed [10]. Yet, soluble carbohydrates have been shown to be insufficient to fully describe the development of freezing tolerance [20]. While sugar levels are often found to positively correlate with freezing tolerance, the underlying regulatory mechanisms are poorly understood. On a whole plant level, it remains elusive whether sugar accumulation may result from reduced sink activity, because growth retardation at low temperatures is stronger than the reduction of photosynthetic activity [21]. Additionally, it is not clear whether sugars function as cryoprotective substances or because they are substrates for the cryoprotectant synthesis [19].

Together with sugars, also pools of organic and amino acids are significantly affected during cold-induced metabolic reprogramming. Aspartate, ornithine and citrulline were found to increase during cold exposure of *Arabidopsis thaliana* indicating the reprogramming of the urea cycle [14]. Beyond, the authors observed a cold-induced increase in levels of alpha-ketoglutarate, fumarate, malate and citrate which they suggested to result from an up-regulation of the citric acid cycle. Although many observations revealed an increase of metabolite levels to be characteristic for cold acclimation, the magnitude of changes in the metabolome does not necessarily indicate the capacity of *Arabidopsis* to increase its freezing tolerance [12]. A prominent example which shows the possible discrepancy between metabolic reprogramming and gain of freezing tolerance is the comparison of the freezing sensitive natural accessions Cvi, which originates from Cape Verde Islands, and C24, originating from the Iberian Peninsula. Both accessions similarly increase their freezing tolerance during cold acclimation while concomitant metabolome changes were found to differ dramatically [3]. It might not be surprising that the coordination of a complex trait like freezing tolerance cannot be directly related to one certain metabolic output, but, simultaneously, this observation indicates a high level of plasticity which is characteristic for intraspecific molecular responses to environmental cues. In this context, most of the naturally occurring biochemical mechanisms and metabolic regulatory strategies to acclimate to low temperature still remain elusive.

Plant growth is significantly reduced due to cold exposure. Although low temperature significantly affects metabolic processes and resource allocation, growth is not necessarily limited by photosynthetic activity. Following a period of 1 to 3 days after exposure to low temperature, during which cold stress is sensed and acclimation is initiated, rates of photosynthetic carbon assimilation can be almost fully recovered [22]. Together with the finding that growth is affected more significantly than photosynthesis during exposure to water deficit [23], this indicates that growth during stress exposure might rather be limited by sinks than sources. Such a cold-induced sink limitation has been discussed to be the reason for the characteristic accumulation of sugars during cold exposure. Although high levels of sugars have been shown to potentially repress the expression of photosynthetic genes [24, 25], cold acclimation and development at low temperature was found to reduce or even fully revert this effect [26–28]. Additionally, cold acclimation was found to have a significant effect on leaf respiration of *Arabidopsis thaliana* [29]. Both respiration rates in the light and in the dark were described to increase significantly during cold acclimation, while the more pronounced effect was found for respiration in darkness. Moreover, although cytosolic hexose phosphate concentrations increased dramatically, there was no significant correlation observed with respiration in the light indicating that respiration is not limited by substrate availability under low temperature stress [29].

Although the above-mentioned findings only represent an excerpt from current findings about growth regulation and cold acclimation strategies in *Arabidopsis*, it clearly indicates a highly complex and interlaced relationship between metabolic and physiological consequences of low temperature. Systems biology focuses on such complex questions and has become a rapidly expanding and attractive research area during the last decade [30]. In a systems biology approach, elements of an interaction network, e.g. a metabolic map, are rather analysed and discussed as interacting components than isolated parts in order to improve the understanding of how a complex biological system is organized and regulated [31].

Research on plant freezing tolerance, growth regulation and plant systems biology has largely been driven by studies in *Arabidopsis thaliana*. The species is native to Europe and central Asia, its biogeography was described in detail, and it was shown that climate on a global scale is sufficient for shaping the range boundaries [32]. When compared to other *Brassicaceae* species, *Arabidopsis* has a wide climatic amplitude and shows a latitudinal range from 68 to 0°N, which makes it suitable for the analysis of variation in adaptive traits [33]. *Arabidopsis* represents a predominantly selfing species, and, hence, most of the individual *Arabidopsis* plants collected in nature represent homozygous inbred lines [34]. These homozygous lines are commonly referred to as accessions, representing genetically distinct natural populations that are specialized to particular sets of environmental conditions. The variation of morphological and physiological phenotypes enables the differentiation of most of the collected *Arabidopsis* accessions from others. In particular, considering the tolerance to abiotic factors, e.g. low temperature, a large variation has been reported (e.g. [33]), making *Arabidopsis* an attractive system to study plant-environment interactions.

In the present study, two of these *Arabidopsis* accessions were analysed with respect to naturally occurring variation in the traits of growth regulation and freezing tolerance. The selection of the two accessions, Cvi (origin: Cape Verde Islands) and Rschew (origin: Western Russia), was based on findings of previous studies which have shown that Cvi represents a freezing sensitive accession while Rsch is freezing tolerant (e.g. [35]). Based on this finding and due to their large distance with respect to geographical origin, cold acclimation capacity and cold-induced gene regulation [3], the molecular and biochemical study of both accessions can be expected to provide a suitable approach to quantify strategies of growth maintenance during environmental fluctuations. As previous work has already indicated, a multi-layered design of molecular physiological studies was necessary in order to derive coherent conclusions on a genome-wide level [11, 36]. Thus, the present study aimed at a comprehensive characterization of metabolomic, proteomic and phosphoproteomic levels of both natural accessions to unravel differential strategies of growth regulation in a changing environment.

## Results

### Differential growth of Cvi and Rsch during cold acclimation

**Fig. 1:**
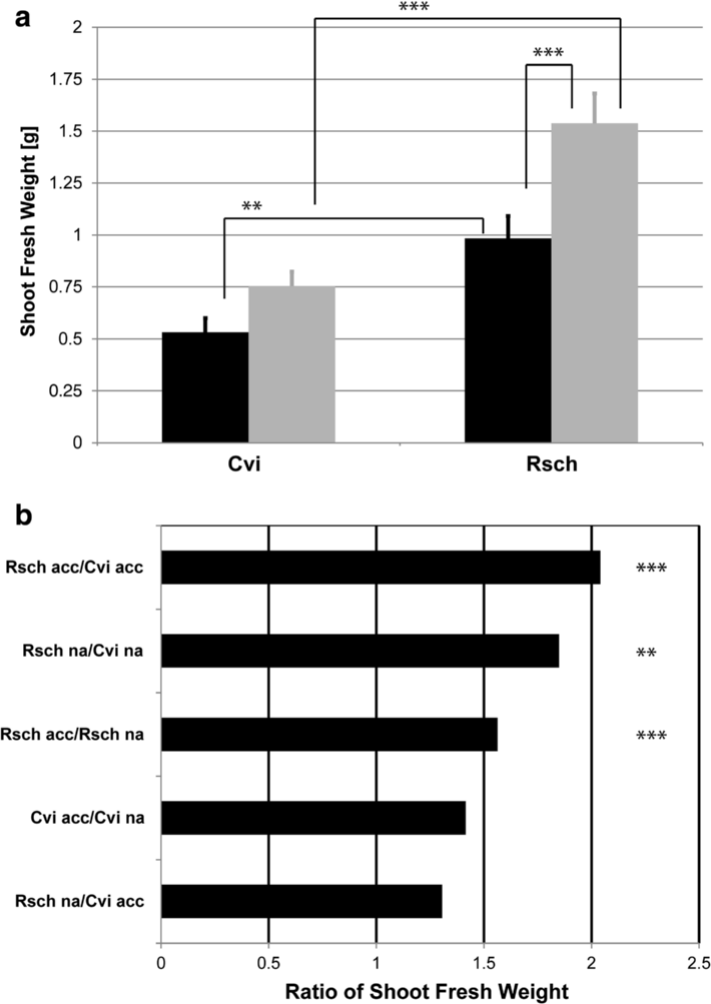
Comparison of shoot fresh weight. **a** Absolute shoot fresh weight of accessions Cvi and Rsch before (na, black bars) and after (acc, grey bars) cold acclimation. Error bars represent means ± SE (*n* = 15). **b** Ratios of mean shoot fresh weights. Asterisks indicate significance tested in an ANOVA (** *p* < 0.01; *** *p* < 0.001)

Growth behaviour of both accessions was characterized by recording the total fresh weight of leaf rosettes from 15 independently grown plants for each acclimation state, i.e. the non-acclimated (na) and acclimated (acc) state (Fig. 1a). Analysis of variance (ANOVA) revealed a significantly higher fresh weight of Rsch plants before (na) and after (acc) cold acclimation compared to Cvi (Fig. 1b). Additionally, plants of the accession Rsch were found to increase their fresh weight significantly (~1.6-fold) during cold acclimation while this was not observed for Cvi (Fig. 1b; Remark: when applying Student’s t-test, the increase in fresh weight of Cvi was detected to be significant; *p* = 0.018). Furthermore, cold acclimated plants of Cvi did not differ in their fresh weight compared to non-acclimated plants of Rsch. Most distinct differences in fresh weight, which we interpreted in terms of an average growth rate [37], were observed between cold acclimated plants of Rsch and Cvi (Ratio >2).

### Integrative profiling of metabolites, proteins and phosphoproteins during cold acclimation

**Fig. 2:**
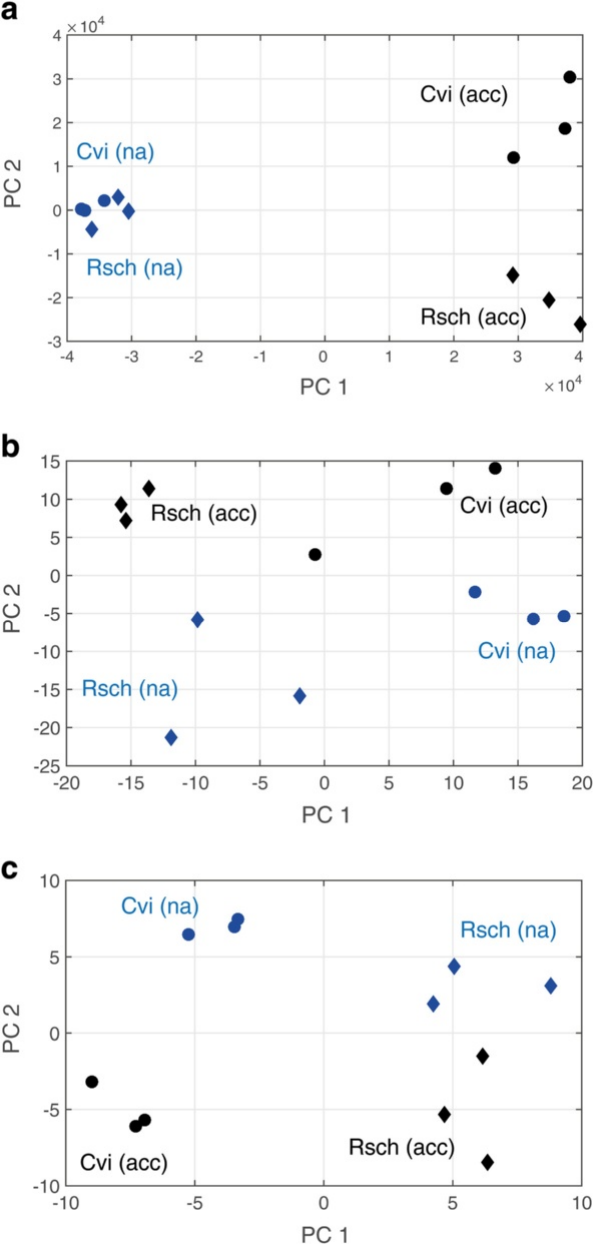
Principal component analysis (PCA) on levels of (**a**) the primary C/N-metabolome, (**b**) protein abundance, and (**c**) phosphopeptide abundance. Accession samples are represented by filled circles (Cvi) and filled diamonds (Rsch). Blue colour indicates non-acclimated samples, black colour indicates acclimated samples. Detailed information about loadings and explained variances of the PCA as well as absolute levels of metabolites, relative levels of proteins and phosphopeptides are provided in the supplements

**Fig. 3:**
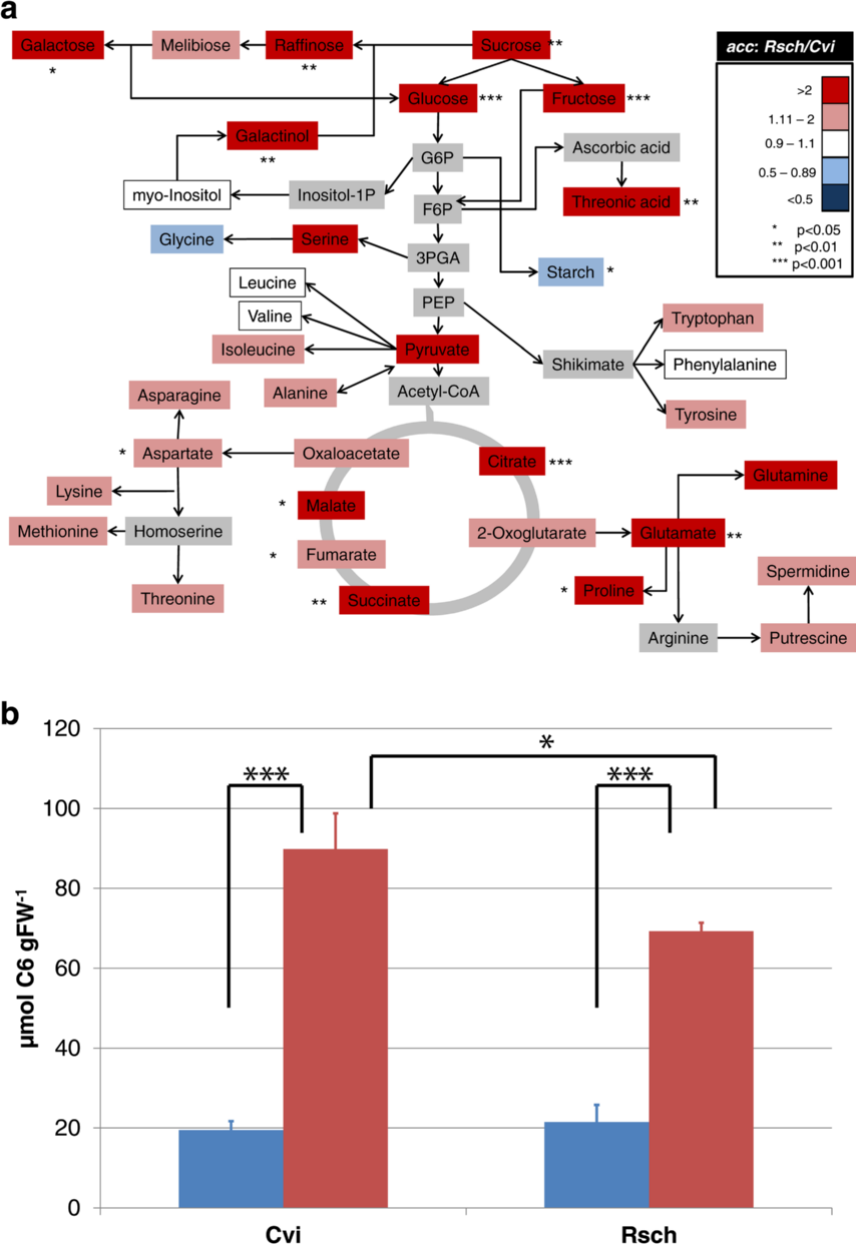
The primary metabolome in cold-acclimated leaf samples of accessions Rsch and Cvi. **a** Ratios of metabolite levels which were built by dividing the absolute mean values of metabolite levels of Rsch by levels of Cvi which were assessed by a GC-TOF/MS measurement (see Methods *-* GC-MS Metabolite Analysis; *n* = 3). Asterisks indicate significant differences as described in the figure. Grey-coloured metabolites were not experimentally analysed. **b** Absolute starch levels in non cold-acclimated (blue bars) and cold acclimated (red bars) leaf samples of Cvi and Rsch (*n* = 3). Asterisks indicate significant differences (* *p* < 0.05; ** *p*<0.01; *** *p* < 0.001)

For a comprehensive molecular characterization of both accessions, the metabolome, proteome and the phosphoproteome, i.e. phosphopeptide abundance, was analysed applying an integrative analytical GC-MS and LC-MS platform [38–43]. Statistical dimensionality reduction by Principal Component Analysis (PCA) revealed a clear separation of both accessions and acclimation states on all levels of molecular organization (Fig. 2). In the non-acclimated state, the accessions were not separated by metabolite profiling including the main components of C/N leaf metabolism. (Fig. 2a). In contrast, after cold-acclimation both accessions were significantly separated (Fig. 2a). Levels of soluble sugars, threonic acid, citrate, succinate, malate, fumarate, glutamate, proline and aspartate were found to be significantly higher in Rsch, while a high level of transitory starch was found to be characteristic for Cvi (Fig. 3a, b; Additional file 1: Table S1; Additional file 2: Figure S1).

**Fig. 4:**
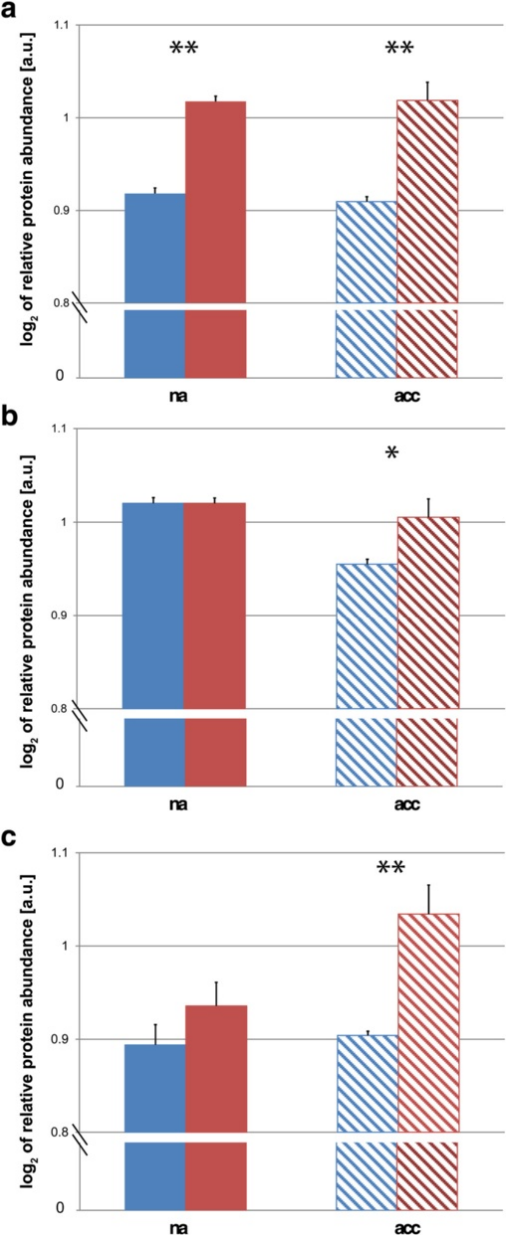
Relative protein levels of amylase enzymes in non cold-acclimated (na) and cold-acclimated (acc) leaf samples. **a** Levels of alpha-amylase-like 3 (AMY3; AT1G69830), and (**b**) Levels of chloroplast beta-amylase (BAM3; AT4G17090), and (**c**) Levels of isoamylase 3 (ISA3; AT4G09020). Blue colour indicates the accession Cvi, red colour indicates the accession Rsch (*n* = 3). Filled bars represent means ± SD of na samples, hatched bars represent means ± SD of acc samples. Asterisks indicate significant differences between accessions (* *p* < 0.05; ** *p* < 0.01). Abundances were normalised to total protein content of the sample

On the proteome level, PCA revealed a clear separation of both accessions and conditions (Fig. 2b). Accessions were separated on PC1 while the acclimation process became visible on PC2. Although the explanatory power of PC1 was only about 8 % higher than that of PC2 (Additional file 3: Figure S2), this indicated that the strongest observable effect in the proteome was due to accession-specific differences followed by changes induced by the cold acclimation process. The strongest observed accession-specific separation in the proteome appeared due to differences in carbohydrate metabolism, amino acid metabolism, abiotic stress-related proteins, protein synthesis and degradation, sulphur assimilation (ATP-sulfurylase, ATP-S), glucosinolate biosynthesis, and redox regulation (Additional file 4: Table S2). Particularly, relative alpha- and beta-amylase enzyme levels, i.e. alpha-amylase-like 3 (AMY3; AT1G69830) and chloroplast beta-amylase (BAM3; AT4G17090), showed a differential pattern in both accessions (Fig. 4). While AMY3-levels were found to be constitutively higher in Rsch (Fig. 4a), levels of BAM3 showed an acclimation-dependent decrease in Cvi (Fig. 4b). Levels of isoamylase 3 (ISA3; AT4G09020) were found to significantly increase during cold acclimation in Rsch while no significant change in ISA3-levels was observed for Cvi (Fig. 4c).

**Fig. 5:**
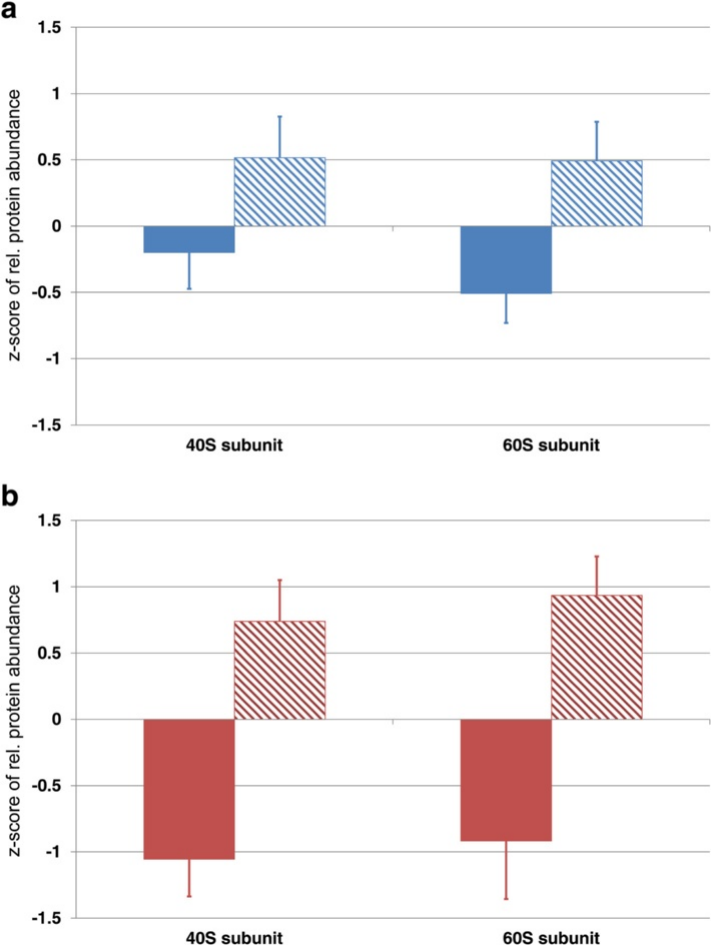
Cold-induced increase of the ribosomal 40S and 60S subunit in the *Arabidopsis* accessions (**a**) Cvi and (**b**) Rsch. Colours indicate the different accessions (blue: Cvi; red: Rsch), filled and hatched bars differentiate cold acclimation states (filled: na; hatched: acc). Bars and error bars represent the mean ± SD of relative protein abundance after standardization (zero mean & unit variance, *z-score*). Means ± SD were built from those ribosomal protein compounds which were identified to contribute strongest to the separation of na and acc samples (see PCA in Fig. 2b and Additional file 4: Table S2; 60S subunit *n* = 11; 40S subunit *n* = 12)

In addition to this accession-specific effect, the cold acclimation process most significantly affected proteins related to processes involved in photosynthetic light reactions and the Calvin cycle (Additional file 4: Table S2). PCA revealed a very pronounced cold acclimation-induced effect for levels of the ribosomal 40 and 60S subunit (see Additional file 4: Table S2) indicating a systematic reprogramming of the translational machinery in both accessions (Fig. 5). A detailed list of ribosomal components is provided in the supplements (Additional file 5: Table S3). In both accessions, levels of several ribosomal protein components were significantly increased after cold acclimation, and this effect was found to be even more pronounced in Rsch than in Cvi (see Additional file 5: Table S3).

A full and detailed list of all functional categories of the proteome and their hierarchy concerning the accession- and acclimation-specific separation is provided in the supplements (Additional file 4: Table S2).

### Changes in the phosphoproteome of Cvi and Rsch during cold acclimation

**Fig. 6:**
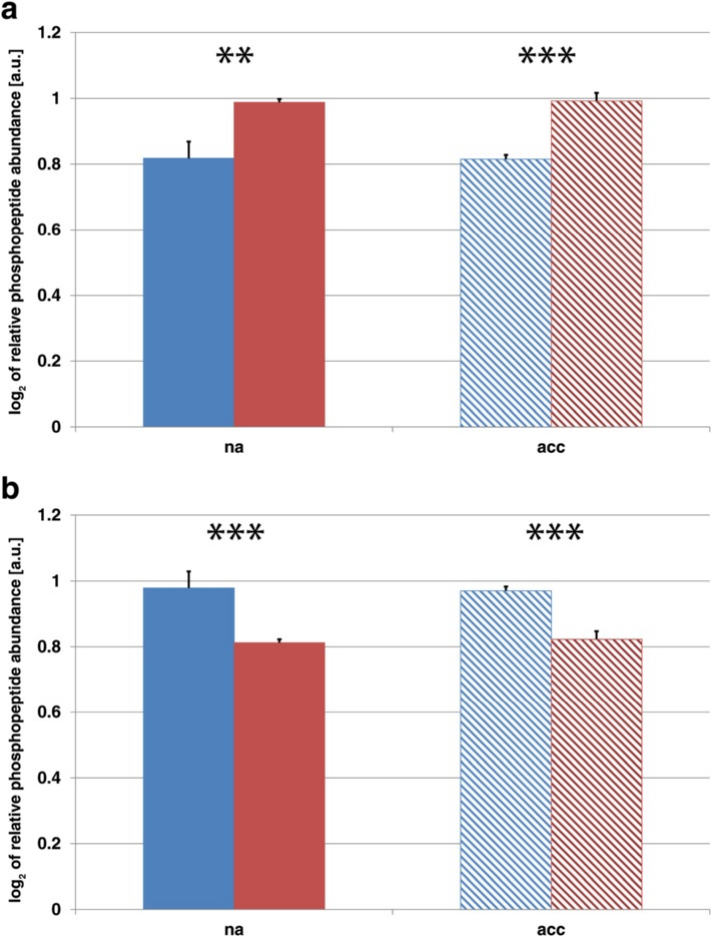
Relative abundance of phosphorylated peptides of (**a**) BASIC PENTACYSTEINE 6, and (**b**) plasma membrane intrinsic protein PIP2;3. Colours indicate samples of the two different accessions Cvi (blue) and Rsch (red) before (na; filled bars) and after (acc; hatched bars) cold acclimation. Bars and error bars represent the mean ± SD of relative phosphopeptide abundance (*n* = 3). Asterisks indicate significant differences (** *p* < 0.01; *** *p* < 0.001)

Similar to the proteome, also the phosphoproteome, i.e. the detected and quantified phosphopeptide abundances, revealed a stronger separation of accessions compared to acclimation states (Fig. 2c, Additional file 3: Figure S2). Yet, also in this context the explained variances by PC1 (accession) and PC2 (acclimation) only differed by ~6 % indicating a similar contribution to the separation. The most dominating accession-specific effects in the phosphoproteome were found to comprise processes of membrane transport and trafficking, modulation of transcription factors and ubiquitination (Additional file 6: Table S4). In particular, one of the most characteristic and significant differences between Cvi and Rsch could be observed for the phosphorylation levels of BASIC PENTACYSTEINE 6 (BPC6; AT5G42520; Fig. 6a), a member of a plant-specific transcription factor family. The phosphorylation level was found to be constitutively higher in Rsch compared to Cvi (*p* < 0.01). In contrast, phosphorylation levels of the plasma membrane intrinsic protein PIP2;3 (AT2G37180) were found to be constitutively higher in Cvi (Fig. 6b; *p* < 0.001).

**Fig. 7:**
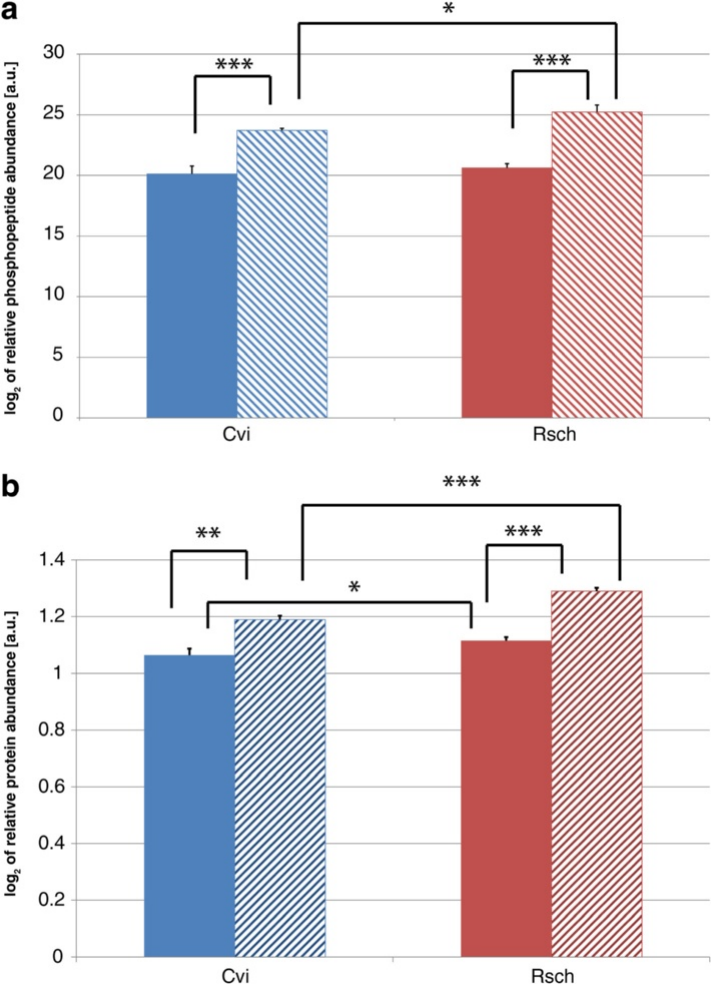
Relative phosphorylation and protein levels of COR78. **a** Bars represent mean values (±SD, *n* = 3) of relative COR78 (AT5G52310) phosphopeptide abundance. **b** Bars represent mean values (±SD, *n* = 3) of relative COR78 (AT5G52310) protein abundance. Colours indicate the accessions (Cvi: blue; Rsch: red). Filled bars indicate values of non-cold acclimated samples, hatched bars indicate values of cold acclimated samples. Asterisks indicate significant differences (* *p* < 0.05, ** *p* <0.01, *** *p* < 0.001)

Detected cold acclimation-induced changes in the phosphoproteome, which were displayed on PC2 (Fig. 2c), revealed a complex pattern of *in vivo* phosphorylation affecting various transcription factors, photosynthetic electron carriers, ribosomal subunits, processes of protein assembly and the cytoskeleton (Additional file 6: Tables S4 and Additional file 7: Table S5). The most significant cold acclimation-induced effect on phosphopeptide levels was detected for the protein Cold Regulated 78, COR78 (AT5G52310). In both accessions, relative levels of phosphorylated COR78 peptides were found to be significantly increased after cold acclimation (*p* < 0.001; Fig. 7a). Further, a significantly higher phosphorylation level was detected in cold acclimated samples of Rsch compared to acclimated samples of Cvi (*p* < 0.05). The same pattern was observed for the relative protein abundance of COR78 which was also significantly higher in non-acclimated samples of Rsch (*p* < 0.05; Fig. 7b).

### Integrative analysis of metabolism and predicted protein-protein-interaction networks (PPIN) during cold acclimation

**Fig. 8:**
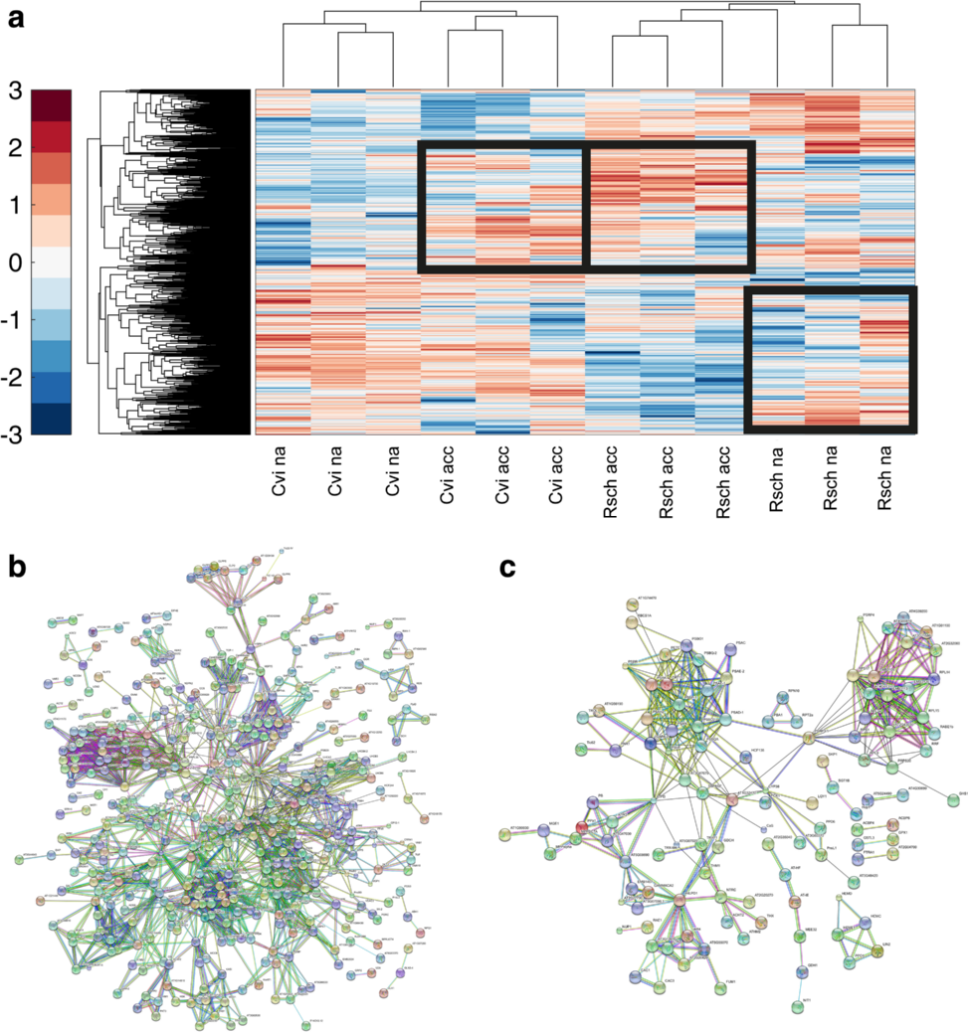
Hierarchical cluster analysis and functional protein interaction networks of cold acclimation-induced reprogramming. **a** Hierarchical clustering of *Arabidopsis* accessions, acclimation states, and metabolite, protein and phosphopeptide abundances based on Euclidean distances. Columns represent non-cold acclimated (na) and cold-acclimated (acc) samples of Rsch and Cvi. Rows represent metabolites, proteins and phosphopeptides. Blue rectangles indicate the characteristic compounds which were chosen for reconstruction of the cold-acclimation induced interaction networks (part (**b**) and (**c**)). **b** Protein-protein interaction network of all proteins and phosphoproteins which were found to be involved in the cold acclimation-induced reprogramming of Rsch. **c** Protein-protein interaction network of all proteins and phosphoproteins which were found to be involved in the cold acclimation-induced reprogramming of Cvi. Interaction networks were created using the STRING database for known and predicted protein-protein interactions (setting: highest confidence (0.9); http://string-db.org/) [85]. A detailed list of protein-protein interactions for both accessions is provided in the supplement (Additional file 9: Table S6 and Additional file 10: Table S7)

To derive a comprehensive overview of accession-specific and cold acclimation-induced molecular processes, collected experimental information about metabolite, protein and phosphopeptide levels was clustered according to their Euclidean distance after standardization (zero mean & unit variance; Fig. 8a). While for both Cvi and Rsch clusters could be identified which were not affected by the cold acclimation process (Additional file 8: Table S8), cold affected proteins were analysed in protein interaction networks predicted by the STRING database (see Methods) (Fig. 8b, c). Both created interaction networks differed clearly in their size. While the cold-response network of the cold-tolerant accession Rsch comprised almost 4000 protein interactions (Additional file 9: Table S6), the Cvi network only comprised about 500 interactions (Additional file 10: Table S7). A predominant and common effect of cold acclimation in both accessions was the reprogramming of protein synthesis, i.e. of ribosomal subunits (Table 1). About 65–80 % of all cold-affected protein interactions were found to be related to this functional category. In a more specific context, this finding is also displayed in Fig. 5 showing the cold-induced reprogramming of the ribosomal 40 and 60S subunit. A more contrasting picture between both accessions was observed for proteins and phosphorylation levels associated with processes of protein degradation, Calvin Cycle, photosynthetic light reactions, TCA cycle, amino acid synthesis, photorespiration, redox metabolism, protein folding, glycolysis, and lipid metabolism (Table 1). These processes were found to be involved much stronger in the cold acclimation responsenetwork of Rsch compared to Cvi.

**Table 1:** Proteomic adjustment and functions in the cold acclimation of Rsch and Cvi

Functional Category	*Arabidopsis* Accession	Relative contribution to accession-specific cold response [%]
Protein synthesis	Rsch	65.59
	Cvi	79.47
Protein degradation	Rsch	5.81
	Cvi	1.83
Calvin cycle	Rsch	4.54
	Cvi	0.34
Light reactions	Rsch	4.01
	Cvi	0.34
TCA	Rsch	2.76
	Cvi	0.57
Amino acid synthesis	Rsch	2.23
	Cvi	1.72
Not assigned	Rsch	1.61
	Cvi	0.92
Photorespiration	Rsch	1.40
	Cvi	-
Redox	Rsch	1.23
	Cvi	0.46
Protein folding	Rsch	1.17
	Cvi	0.57
Glycolysis	Rsch	1.00
	Cvi	0.23
Lipid metabolism	Rsch	1.00
	Cvi	0.23
Nucleotide metabolism	Rsch	0.95
	Cvi	-
OPP	Rsch	0.91
	Cvi	-
Stress	Rsch	0.72
	Cvi	5.50
Gluconeogenesis	Rsch	0.65
	Cvi	-
Secondary metabolism	Rsch	0.61
	Cvi	0.23
S-assimilation	Rsch	0.40
	Cvi	-
Protein targeting	Rsch	0.33
	Cvi	0.46
mETC	Rsch	0.32
	Cvi	0.46
DNA synthesis	Rsch	0.27
	Cvi	-
C1 metabolism	Rsch	0.26
	Cvi	-
Major CHO metabolism	Rsch	0.26
	Cvi	-
Tetrapyrrole synthesis	Rsch	0.26
	Cvi	1.61
Transport	Rsch	0.23
	Cvi	-
Misc.	Rsch	0.20
	Cvi	-
N-Metabolism	Rsch	0.20
	Cvi	-
Co-factor and vitamin metabolism	Rsch	0.13
	Cvi	0.69
Signalling	Rsch	0.13
	Cvi	0.34
Amino acid degradation	Rsch	0.12
	Cvi	-
Minor CHO metabolism	Rsch	0.12
	Cvi	-
Cell cycle	Rsch	0.09
	Cvi	-
Protein assembly/Cofactor ligation	Rsch	0.09
	Cvi	0.11
RNA	Rsch	0.09
	Cvi	3.56
Protein PTM	Rsch	0.07
	Cvi	-
Biodegradation of xenobiotics	Rsch	0.06
	Cvi	-
Protein activation	Rsch	0.06
	Cvi	0.23
Hormone metabolism	Rsch	0.04
	Cvi	-
Cell organisation	Rsch	0.03
	Cvi	-
Metal handling	Rsch	0.03
	Cvi	-
Cell division	Rsch	0.01
	Cvi	-

All listed categories represent protein functions and their relative portion to all interactions which were identified by the STRING database analysis and which were found to be affected after the cold acclimation process (see Fig. 8)

## Discussion

Cold acclimation of plants represents a multifaceted and multigenic process affecting various levels of molecular organisation, e.g. gene expression, RNA processing or post-translational regulation [44, 45]. Hence, although numerous comprehensive studies have unravelled many crucial processes being involved in the acclimation process (for an overview see e.g. [46]), it is not surprising that many gaps still exist in our understanding of how metabolism is reprogrammed, and how the metabolic output is linked to the observed physiological output, e.g. changes in growth and yield. In general, plant growth requires a sufficient supply with energy, water and nutrients and is regulated in response to environmental changes. These environmental cues are sensed and integrated by a highly complex and conserved signalling network [47].

An efficient balancing of photosynthesis and respiration was shown to be a prerequisite for plant growth [48] and cold acclimation [29]. With regard to these two central processes, our findings revealed a more complex cold-induced metabolic reprogramming in the cold tolerant *Arabidopsis* accession Rsch which also showed a significantly higher shoot fresh weight than both non-acclimated and acclimated plants of Cvi (see Fig. 1). In addition, also glycolysis, TCA cycle and pathways of amino acid biosynthesis were found to be differentially affected by low temperature in both accessions. Together with the observed levels of sugars, organic and amino acids, which were, on an average, significantly higher in acclimated plants of Rsch, this points to a differential cold-induced redirection of carbon equivalents in both accessions. While we cannot experimentally exclude a limitation of CO_2_ uptake as a reason for the lower metabolite levels in cold-acclimated plants of Cvi, there are several indications which rather suggest a differential regulation of carbon allocation to be the reason for the observed phenotype. First, on the level of the total proteome, we could observe a separation of acclimation states but not of accessions by cold-induced protein dynamics related to photosynthetic dark and light reactions (Additional file 11: Table S9). Second, in a former study, the analysis of the photosynthetic carbon uptake was found to be similar in cold-acclimated plants of cold sensitive and tolerant accessions [49]. While Nägele and colleagues did not analyse the Cape Verde accession Cvi but the cold-sensitive accession C24 originating from the Iberian Peninsula, further support of this hypothesis is provided by another study in which photosynthetic acclimation of Cvi was compared to the Finnish accession Hel-1, originating from Helsinki [50]. There, the author found that both accessions, originating from contrasting climates, showed a highly similar capability to acclimate to a broad regime of temperature and irradiance. Another indication for a non-limited CO_2_-uptake is provided by the starch levels which were found to increase to a significantly higher level in Cvi than in Rsch (see Fig. 3). This agrees with the findings of Guy and co-workers who also described a significantly higher starch level in Cvi compared to Rsch after cold acclimation [12]. Based on this observation, Guy and co-workers suggested that, following a sufficiently long acclimation period, even in poorly acclimating accessions like Cvi energy constraints do not seem to limit the acquisition of freezing tolerance. Although our growth conditions (5 °C/7d of acclimation/125 μmol m^−2^ s^−1^) do not exactly reflect the growth conditions applied in the study of Guy and co-workers (4 °C/14d acclimation/90 μmol m^−2^ s^−1^), we still observed a similar output of starch metabolism.

To derive an explanation for the observed differences in starch metabolism, which has previously been suggested to be a major regulator of plant growth [51], the regulation of both starch synthesis and degradation has to be considered. While our study does not account for enzymatic activity, our proteomic results provide evidence for a different regulation of starch metabolism in cold acclimated plants of Cvi and Rsch. While, independently from cold exposure, levels of alpha-amylase AMY3 were found to be constitutively higher in Rsch than in Cvi, a cold-induced significant reduction in the level of beta-amylase BAM3 could only be observed for Cvi, while isoamylase 3, ISA3, was significantly increased only in cold-acclimated plants of Rsch. Alpha-, beta- and isoamylases play crucial roles in starch degradation [52–54], and, hence, these findings hint towards a distinct regulation of starch degradation which was previously discussed to play a decisive role in the process of cold acclimation [55, 56]. Starch molecules consist of mostly unbranched amylose (alpha-1,4-linked glucosyl moieties) and branched amylopectin (alpha-1,6-linked moieties). While alpha-amylase, hydrolysing the alpha-1,4-glucosidic linkages of starch, plays a central role in the degradation of storage starch in endosperm of germinating cereal seeds [57], a disruption of AtAMY3 by insertional mutagenesis did not affect starch degradation in *Arabidopsis* leaves [58]. However, removal of AMY3 in addition to the debranching, alpha-1,6-linkage hydrolysing, enzyme ISA3 was shown to lead to a strong starch excess phenotype [54]. A triple mutant with an additional removal of limit dextrinase, LDA, which represents another debranching enzyme, was finally shown to result in an effective block of starch breakdown accumulating even higher levels of starch than observed before in the double mutant [54]. While our presented shotgun proteomics approach could not resolve the cold-induced effect on LDA in either of both accessions, our findings indicate that the combination of constantly lower AMY3-levels in Cvi and a cold-induced increase in ISA3-levels in Rsch might provide an explanation for the higher starch levels observed in cold-acclimated plants of Cvi.

The complete process of (transitory) starch breakdown from the insoluble granule to the soluble compounds maltose and glucose comprises numerous additional steps and classes of enzymes, finally resulting in a complex and tightly (redox) regulated pathway [52, 59]. Beta-amylases (BAMs) primarily hydrolyse glucan chains, which have been previously released and linearized, liberating maltose [52]. The multigene family of BAMs in *Arabidopsis thaliana* comprises nine genes, and *BAM3* was shown to encode a catalytically active plastidial enzyme playing a central role in leaf starch degradation at night in mesophyll cells [60, 61]. Hence, our finding of a significant decrease of BAM3 protein levels in cold acclimated plants of Cvi provides a further explanation for the strong increase of starch levels. The observation of a decrease in BAM3 protein levels contrasts the finding of a cold induced increase of *BAM3* expression [56]. However, in a recent publication Monroe and co-workers derived a more complex picture in which the authors observed a decline in BAM3 activity after 2d and 4d of cold stress while *BAM3* mRNA levels clearly increased [62]. Although these results were derived from studies within the genetic background of the Arabidopsis accession Columbia-0, and, hence, might not directly be comparable to the background of Cvi, they indicate the complex interplay of molecular levels of organization during exposure to a fluctuating environment. Such an adaptive and differential regulation of starch metabolism in response to cold was also exemplified in a previous study on the starchless *Arabidopsis thaliana pgm* mutant being deficient in a phosho-glucomutase activity [63]. In this study, the cold/heat-stress-induced increase of raffinose-family-oligosaccharide levels in the *pgm* mutant plants revealed an unexpected flexibility to adjust central metabolism to temperature stress in the absence of transitory starch.

Based on our investigation of the two natural accessions Rsch and Cvi during cold-acclimation, we suggest that the orchestration of growth and cold acclimation differs significantly in the redirection of photoassimilates between soluble metabolic compounds and the insoluble storage compound starch. In addition, the observation described in a previous study, that the biomass formation in the starchless *pgm* mutant is restricted by high respiratory losses in the root [48], allows us to hypothesise that the differences we observed in the fresh weight of Cvi and Rsch might also be due to a differential regulation of sink-source interaction both before and after the cold acclimation period. In future studies it would be interesting to analyse whether the observed differences in starch degradation are somehow related to resource allocation and root respiration in both accessions.

In context of *Arabidopsis* cold acclimation, the C-repeat binding factor (CBF) pathway belongs to one of the most intensively studied pathways which has a crucial role in the development of freezing tolerance [64]. Within minutes after transfer to low temperature, the CBF1-3 [65], i.e. DREB1a-c [66], expression is induced. They encode members of the AP2/ERF family of transcription factors recognizing the C-repeat (CRT)/dehydration-responsive element (DRE) being present in the promotors of CBF-targeted genes [66]. The constitutive overexpression of either CBF1, 2 or 3 alters the expression of cold-regulated (COR) genes resulting in an increase of freezing tolerance without exposure to low temperature [67, 68]. In the present study, the level of COR78 (AT5G52310) and its phosphorylation were observed to be positively correlated with the acclimation state of both accessions. Further, independent from the acclimation state, protein levels were found to be constitutively higher in Rsch than in Cvi. Interestingly, COR78 transcript abundance was previously discussed to be regulated by sucrose [69] which would explain our findings of higher protein abundance and sucrose levels in Rsch (see Figs. 3 and 7). In addition, these observations allow for the speculation about a link between sugar signalling networks and the cold responsive gene regulation which could probably comprise central conserved signalling compounds like the complex and antagonistic interaction network spanned by the kinases Sucrose-non-fermenting-1-Related Protein Kinase 1(SnRK1) and Target Of Rapamycin (TOR) [70].

Finally, the observation of differentially phosphorylated transcription factors, like the BASIC PENTACYSTEINE (BPC), but also membrane proteins, e.g. PIP2;3 aquaporins which are involved in numerous developmental and growth-regulatory processes [71, 72], clearly shows the wide range of cellular processes which might contribute to a systematic and differential stress acclimation output in naturally occurring accessions of Arabidopsis. Our results indicate that a comprehensive reprogramming not only of the process of protein synthesis, but also of metabolic pathways regulating the flux of photoassimilates to the TCA cycle and to pathways of amino acid biosynthesis, contributes to the stabilization of a metabolic homeostasis during cold acclimation. Together with previous studies on the stress-induced dynamics of protein phosphorylation patterns, which have, for example, revealed the central role of protein phosphorylation in cold-induced subcellular sugar allocation [73], and its applicability to crop science [74], this clearly indicates the necessity for integrative molecular profiling approaches to unravel a comprehensive picture of complex plant acclimation strategies.

## Conclusions

The findings presented in this study provide evidence for a central role of the starch degradation pathway in the molecular orchestration of plant growth and abiotic plant-environment interactions in different natural Arabidopsis accessions. We conclude that manipulation of the starch degradation pathway represents a promising target for improving plant yield and stress tolerance. We hypothesise that stress-induced reprogramming of starch degradation plays a central role in the orchestration of photosynthetic metabolism rather than being a pure consequence from cold-induced metabolic changes. Together with reprogramming of translational regulation and protein synthesis it seems to differentially affect the cold-induced metabolic homeostasis which finally contributes to the observed acclimation output.

## Methods

### Plant cultivation and sampling strategy

Plants of *Arabidopsis thaliana* natural accessions Cvi-0 (NASC ID: N1097) and Rsch-0 (NASC ID: N1490; both accessions donated by: Albert Kranz Institute for Molecular Biosciences, Department of Biological Sciences, Johann Wolfgang Goethe-Universität Frankfurt am Main) were cultivated in a growth chamber under controlled conditions. The substrate for plant growth was composed of Einheitserde® ED63 and perlite. Plants were watered daily and fertilized once with NPK fertilization solution (WUXAL®Super; MANNA°-Dünger, Ammerbuch). Light intensity was 75 μmol m^−2^ s^−1^ in a 8/16 h day/night cycle with a relative humidity of 70 % and a temperature of 22 °C/16 °C. 28 days after sowing, light intensity was increased to 125 μmol m^−2^ s^−1^ in a 16/8 h day/night cycle. At bolting stage, which was 43 days after sowing, samples of non-acclimated plants were collected from both accessions at midday, i.e. 8 h after light on. One sample consisted of 3 leaf rosettes. Non-sampled plants were transferred to 5 °C at 125 μmol m^−2^ s^−1^ in a 16/8 h day/night cycle with 70 % humidity. After 7 days at 5 °C, leaf rosettes were sampled as described for non-acclimated plants, i.e. each sample consisted of 3 leaf rosettes. At this growth stage, both accessions had induced inflorescence which was slightly higher (<1 cm) in Cvi than in Rsch. All samples were immediately quenched in liquid nitrogen. Sample material was stored at −80 °C until use.

### GC-MS metabolite analysis

Frozen sample rosettes were ground to a fine powder with pestle and mortar under frequent cooling with liquid nitrogen. Polar metabolites were extracted and chemically derivatized as described previously [75, 76]. Gas chromatography coupled to mass spectrometry (GC-MS) analysis was performed on an Agilent 6890 gas chromatograph (Agilent Technologies®, Santa Clara, CA, USA) coupled to a LECO Pegasus® 4D GCxGC-TOF mass spectrometer (LECO Corporation, St. Joseph, MI, USA). Compounds were separated on an Agilent HP5MS column (length: 30 m, diameter: 0.25 mm, film: 0.25 μm). Deconvolution of the total ion chromatograms was performed using the LECO Chromatof® software. For absolute quantification of metabolites, peak areas were compared to calibration curves within a linear range of detection. Compound names, retention indices and mass-charge (m/z)-ratios which were used for peak quantification are provided in the supplements (Additional file 12: Table S10).

### Protein extraction, phosphopeptide enrichment and LC-MS analysis

Total protein was extracted from 1 g of ground plant material as previously described [77]. Protein pellets were dissolved in 8 M urea/100 mM ammonium bicarbonate (AmBic) and protein concentration was determined with the Bio-Rad Bradford Assay using BSA as a standard. 1050 μg of total protein per sample were first reduced with dithiothreitol (DTT) at concentration of 5 mM at 37 °C for 45 min. Cysteine residues were alkylated with 10 mM iodoacetamide (IAA) in darkness at room temperature (RT) for 60 min. Alkylation was stopped by increasing DTT concentration to 10 mM and incubating in the dark at RT for 15 min. Proteins were first pre-digested with Lys-C (1:1000 w:w) at 30 °C for 5 h. Then the urea concentration was diluted to 2 M with 50 mM AmBic/10 % acetonitrile (ACN).CaCl_2_ was added to a final concentration of 2 mM. Trypsin digestion (Poroszyme immobilized trypsin; 1:100 v:w) was performed at 37 °C overnight. Protein digests were desalted with C18 extraction materials (Agilent Technologies, Santa Clara, USA) and carbon graphite solid phase extraction (SPE) materials as described elsewhere [78]. After both SPEs, corresponding eluates were pooled, split in two tubes (50 μg for total proteomics and 1000 μg for phosphopeptide enrichment) and dried in a vacuum concentrator. Phosphopeptide enrichment was performed using 10 mg of TiO_2_ (Glygen Corp.) as described previously [40, 79].

One microgram of total protein was separated on a PepMap RSLC 75 μm × 50 cm column (Thermo Fisher Scientific Inc., Waltham, USA) using a 120 min linear gradient from 2 to 40 % of mobile phase B (mobile phase A: 0.1 % [v/v] formic acid (FA) in water; mobile phase B: 0.1 % [v/v] FA in 90 % [v/v] ACN) with 300 nL/min flow rate. MS analysis was done with an Orbitrap Elite instrument (Thermo Fisher Scientific Inc., Waltham, USA) using a data-dependent acquisition method. Precursor masses at range 350–1800 Th were measured in the Orbitrap mass analyser with a resolution of 120 000, 1 × 10^6^ ion population, and 200 ms injection time. MS/MS analysis was done in the linear ion trap with CID fragmentation and rapid scan mode for the 20 most intense ions. Prediction of ion injection time was enabled and the trap was set to gather 5 × 10^3^ ions for up to 50 ms. Dynamic exclusion was enabled with repeat duration of 30 s, exclusion list size was set to 500 and exclusion duration to 60 s.

Phosphopeptides were dissolved in 10 μL of 5 % ACN/0.5 % FA and 5 μL were loaded on the column. The LC-MS analysis was done as the analysis of total protein digest with a few modifications. The gradient was 150 min from 2 to 40 % of mobile phase B and multistage activation was enabled with neural losses of 24.49, 32.66, 48.999, 97.97, 195.94, and 293.91 Da for the 10 most intense precursor ions. Further information about LC-MS analysis for reproducibility of experiments is provided in the supplements (Additional file 13: Table S11).

### Data analysis and statistics

Peptide identification, phosphosite mapping as well as protein and phosphopeptide quantification were performed with MaxQuant 1.4 (http://www.maxquant.org ) [80] and the Andromeda search algorithm [81] against the TAIR10 protein database. Total proteomics analysis was done with the following settings: maximum 2 missed cleavages, methionine oxidation, and protein N-terminal acetylation as dynamic modifications were allowed. Mass tolerance for precursors was set to 5 ppm and for fragment masses to 0.8 Da. The maximum FDR was set to 1 % for both peptide and protein levels. Protein quantification was done with a peptide ratio count of, at least, 2. Phosphopeptide identification was performed applying similar settings as in the total protein analysis. Phosphorylation of serine, threonine and tyrosine residues were additionally allowed to occur as dynamic modifications. Because the phosphorylation near a tryptic site could hinder digestion, 3 missed cleavages were allowed. Quantification was done at peptide level. Further data processing was done with the Perseus 1.5 software. Total proteomics data was log_2_ transformed and filtered so that at least in one of the four conditions all values were present. Data was normalized to median of each sample and missing values were replaced with random numbers drawn from normal distribution of each sample. Phosphoproteomics data was handled similarly but additional filtering steps were applied: only phosphopeptides belonging to category I (localization probability >0.75 and score difference >5) [82] were considered for further analyses.

Data evaluation, normalisation and transformation was performed in Microsoft Excel® (http://www.microsoft.com). For Principal Component Analysis (PCA) and hierarchical cluster analysis, z-scores (zero mean, unit variance) were calculated for relative protein and phosphopeptide abundance. Metabolite PCA was performed on absolute levels. Analysis of variance (ANOVA) and Student’s t-test were performed with the R software (The R Project for Statistical Computing; http://www.r-project.org/) (R Core [83]). PCA and hierarchical cluster analysis was performed within the numerical software environment Matlab® (V8.4.0 R2014b; www.mathworks.com) and the toolbox COVAIN [84]. Protein-protein interaction networks were created using the STRING database for Known and Predicted Protein-Protein Interactions (setting: highest confidence, 0.900; http://string-db.org/) (von Mering et al. [85]).

### Availability of supporting data

All supporting data are included as additional files.

## Additional files

Additional file 1:
**Table S1.** Loadings of the metabolite PCA shown in Fig. 2a of the main document. (XLS 38 kb) Additional file 2:
**Figure S1.** Comparison of metabolite levels between non-acclimated and acclimated plants. Ratios were built by dividing the absolute mean values of metabolite levels of Rsch by levels of Cvi, or by dividing absolute mean values of metabolites of acc by na plants. Asterisks indicate significant differences as described in the figure. Grey-coloured metabolites were not experimentally analysed. (TIF 1649 kb) Additional file 3:
**Figure S2.** Explained variances of principal components describing the separation by metabolites, protein and phosphoproteins in Fig. 2 of the main document. (TIF 692 kb) Additional file 4:
**Table S2.** Loadings of the protein PCA shown in Fig. 2b of the main document. (XLS 241 kb) Additional file 5:
**Table S3.** Cold-induced effects on protein levels of components of the 40S and 60S ribosomal subunits. (XLSX 9 kb) Additional file 6:
**Table S4.** Loadings of the phosphoprotein PCA shown in Fig. 2c of the main document. (XLS 66 kb) Additional file 7:
**Table S5.** Normalised experimental data of metabolite, protein and phosphopeptide levels. (XLSX 1210 kb) Additional file 8:
**Table S8.** Proteomic and phosphoproteomic components of Cvi and Rsch which were not included in the cold-induced protein-protein interaction network analysis of Fig. 8. (XLSX 34 kb) Additional file 9:
**Table S6.** Components of the acclimation-induced protein-protein interaction network of Rsch shown in Fig. 8b of the main document. (XLSX 392 kb) Additional file 10:
**Table S7.** Components of the acclimation-induced protein-protein interaction network of Cvi shown in Fig. 8c of the main document. (XLSX 57 kb) Additional file 11:
**Table S9.** Protein dynamics related to photosynthetic dark and light reactions. (XLSX 34 kb) Additional file 12:
**Table S10.** List of metabolic components, retentions indices and mass-to-charge ratios which were applied for absolute quantification of metabolites. (XLSX 11 kb) Additional file 13:
**Table S11.** Proteomics minimal information for reproducibility of experiments. (XLSX 11 kb)

## References

[1] Cramer GR, Urano K, Delrot S, Pezzotti M, Shinozaki K (2011). Effects of abiotic stress on plants: a systems biology perspective. BMC Plant Biol.

[2] Weigel D (2012). Natural variation in Arabidopsis: from molecular genetics to ecological genomics. Plant Physiol.

[3] Hannah MA, Wiese D, Freund S, Fiehn O, Heyer AG, Hincha DK (2006). Natural genetic variation of freezing tolerance in Arabidopsis. Plant Physiol.

[4] Hannah MA, Heyer AG, Hincha DK (2005). A global survey of gene regulation during cold acclimation in Arabidopsis thaliana. PLoS Genet.

[5] Juenger TE. Natural variation and genetic constraints on drought tolerance. Curr Opin Plant Biol. 2013. 10.1016/j.pbi.2013.02.001 23462639

[6] Ikram S, Bedu M, Daniel-Vedele F, Chaillou S, Chardon F (2012). Natural variation of Arabidopsis response to nitrogen availability. J Exp Bot.

[7] Clauw P, Coppens F, De Beuf K, Dhondt S, Van Daele T, Maleux K, et al. Leaf Responses to Mild Drought Stress in Natural Variants of Arabidopsis thaliana. Plant Physiol. 2015. 10.1104/pp.114.254284 PMC4348775 25604532

[8] Nägele T, Heyer AG (2013). Approximating subcellular organisation of carbohydrate metabolism during cold acclimation in different natural accessions of Arabidopsis thaliana. New Phytol.

[9] Samis KE, Murren CJ, Bossdorf O, Donohue K, Fenster CB, Malmberg RL (2012). Longitudinal trends in climate drive flowering time clines in North American Arabidopsis thaliana. Ecol Evol.

[10] Xin Z, Browse J (2000). Cold comfort farm: the acclimation of plants to freezing temperatures. Plant Cell Environ.

[11] Kosova K, Vitamvas P, Prasil IT, Renaut J (2011). Plant proteome changes under abiotic stress--contribution of proteomics studies to understanding plant stress response. J Proteome.

[12] Guy C, Kaplan F, Kopka J, Selbig J, Hincha DK (2008). Metabolomics of temperature stress. Physiol Plant.

[13] Stitt M, Hurry V (2002). A plant for all seasons: alterations in photosynthetic carbon metabolism during cold acclimation in Arabidopsis. Curr Opin Plant Biol.

[14] Cook D, Fowler S, Fiehn O, Thomashow MF (2004). A prominent role for the CBF cold response pathway in configuring the low-temperature metabolome of Arabidopsis. Proc Natl Acad Sci U S A.

[15] Maruyama K, Takeda M, Kidokoro S, Yamada K, Sakuma Y, Urano K (2009). Metabolic pathways involved in cold acclimation identified by integrated analysis of metabolites and transcripts regulated by DREB1A and DREB2A. Plant Physiol.

[16] Mikkelsen MD, Thomashow MF. A role for circadian evening elements in cold-regulated gene expression in Arabidopsis. Plant J. 2009. 10.1111/j.1365-313X.2009.03957.x 19566593

[17] Davey MP, Woodward FI, Quick WP (2009). Intraspecfic variation in cold-temperature metabolic phenotypes of Arabidopsis lyrata ssp. petraea. Metabolomics.

[18] Scarth GW, Levitt J (1937). The frost-hardening mechanism of plant cells. Plant Physiol.

[19] Klotke J, Kopka J, Gatzke N, Heyer AG (2004). Impact of soluble sugar concentrations on the acquisition of freezing tolerance in accessions of Arabidopsis thaliana with contrasting cold adaptation - evidence for a role of raffinose in cold acclimation. Plant Cell Environ.

[20] Hincha DK, Sonnewald U, Willmitzer L, Schmitt JM (1996). The role of sugar accumulation in leaf frost hardiness: investigations with transgenic tobacco expressing a bacterial pyrophosphatase or a yeast invertase gene. J Plant Physiol.

[21] Huner NPA, Öquist G, Sarhan F (1998). Energy balance and acclimation to light and cold. Trends Plant Sci.

[22] Nägele T, Kandel BA, Frana S, Meissner M, Heyer AG (2011). A systems biology approach for the analysis of carbohydrate dynamics during acclimation to low temperature in Arabidopsis thaliana. FEBS J.

[23] Muller B, Pantin F, Genard M, Turc O, Freixes S, Piques M (2011). Water deficits uncouple growth from photosynthesis, increase C content, and modify the relationships between C and growth in sink organs. J Exp Bot.

[24] Goldschmidt EE, Huber SC (1992). Regulation of photosynthesis by End-product accumulation in leaves of plants storing starch, sucrose, and hexose sugars. Plant Physiol.

[25] Sheen J (1994). Feedback control of gene expression. Photosynth Res.

[26] Savitch LV, Barker-Astrom J, Ivanov AG, Hurry V, Oquist G, Huner NP (2001). Cold acclimation of Arabidopsis thaliana results in incomplete recovery of photosynthetic capacity, associated with an increased reduction of the chloroplast stroma. Planta.

[27] Strand A, Hurry V, Gustafsson P, Gardeström P (1997). Development of Arabidopsis thaliana leaves at low temperatures releases the suppression of photosynthesis and photosynthetic gene expression despite the accumulation of soluble carbohydrates. Plant J.

[28] Masclaux-Daubresse C, Purdy S, Lemaitre T, Pourtau N, Taconnat L, Renou J-P (2007). Genetic variation suggests interaction between cold acclimation and metabolic regulation of leaf senescence. Plant Physiol.

[29] Talts P, Parnik T, Gardestrom P, Keerberg O (2004). Respiratory acclimation in Arabidopsis thaliana leaves at low temperature. J Plant Physiol.

[30] Weckwerth W (2011). Green systems biology - From single genomes, proteomes and metabolomes to ecosystems research and biotechnology. J Proteomics.

[31] van Norman JM, Benfey PN (2009). Arabidopsis thaliana as a model organism in systems biology. Wiley Interdiscip Rev Syst Biol Med.

[32] Hoffmann MH (2002). Biogeography of Arabidopsis thaliana (L.) heynh. (brassicaceae). J Biogeography.

[33] Koornneef M, Alonso-Blanco C, Vreugdenhil D (2004). Naturally occurring genetic variation in Arabidopsis thaliana. Annu Rev Plant Biol.

[34] Lawrence MJ (1976). Variations in natural populations of Arabidopsis thaliana (L.) Heynh.

[35] Mishra A, Mishra KB, Höermiller II, Heyer AG, Nedbal L (2011). Chlorophyll fluorescence emission as a reporter on cold tolerance in Arabidopsis thaliana accessions. Plant Signal Behav.

[36] Brunetti C, George RM, Tattini M, Field K, Davey MP (2013). Metabolomics in plant environmental physiology. J Exp Bot.

[37] Chen D, Neumann K, Friedel S, Kilian B, Chen M, Altmann T (2014). Dissecting the phenotypic components of crop plant growth and drought responses based on high-throughput image analysis. Plant Cell Online.

[38] Weckwerth W (2011). Green systems biology—from single genomes, proteomes and metabolomes to ecosystems research and biotechnology. J Proteome.

[39] Beckers GJ, Hoehenwarter W, Rohrig H, Conrath U, Weckwerth W (2014). Tandem metal-oxide affinity chromatography for enhanced depth of phosphoproteome analysis. Methods Mol Biol.

[40] Chen Y, Hoehenwarter W, Weckwerth W (2010). Comparative analysis of phytohormone responsive phosphoproteins in Arabidopsis thaliana using TiO2phosphopeptide enrichment and mass accuracy precursor alignment. Plant J.

[41] Weckwerth W (2011). Unpredictability of metabolism—the key role of metabolomics science in combination with next-generation genome sequencing. Anal Bioanal Chem.

[42] Weckwerth W, Wienkoop S, Hoehenwarter W, Egelhofer V, Sun X (2014). From proteomics to systems biology: MAPA, MASS WESTERN, PROMEX, and COVAIN as a user-oriented platform.

[43] Morgenthal K, Wienkoop S, Scholz M, Selbig J, Weckwerth W (2005). Correlative GC-TOF-MS-based metabolite profiling and LC-MS-based protein profiling reveal time-related systemic regulation of metabolite–protein networks and improve pattern recognition for multiple biomarker selection. Metabolomics.

[44] Barrero-Gil J, Salinas J (2013). Post-translational regulation of cold acclimation response. Plant Sci.

[45] Zhu J, Dong CH, Zhu JK (2007). Interplay between cold-responsive gene regulation, metabolism and RNA processing during plant cold acclimation. Curr Opin Plant Biol.

[46] Hincha DK, Zuther E (2014). Plant cold acclimation and freezing tolerance. Methods Mol Biol.

[47] Tomé FS, Nägele T, Adamo M, Garg A, Marco-llorca C, Nukarinen E (2014). The low energy signaling network. Front Plant Sci.

[48] Brauner K, Hormiller I, Nägele T, Heyer AG (2014). Exaggerated root respiration accounts for growth retardation in a starchless mutant of Arabidopsis thaliana. Plant J.

[49] Nägele T, Stutz S, Hörmiller II, Heyer AG (2012). Identification of a metabolic bottleneck for cold acclimation in Arabidopsis thaliana. Plant J.

[50] Pons T (2012). Interaction of temperature and irradiance effects on photosynthetic acclimation in two accessions of Arabidopsis thaliana. Photosynth Res.

[51] Sulpice R, Pyl E-T, Ishihara H, Trenkamp S, Steinfath M, Witucka-Wall H (2009). Starch as a major integrator in the regulation of plant growth. Proc Natl Acad Sci U S A.

[52] Smith AM, Zeeman SC, Smith SM (2005). Starch degradation. Annu Rev Plant Biol.

[53] Zeeman SC, Smith SM, Smith AM (2007). The diurnal metabolism of leaf starch. Biochem J.

[54] Streb S, Eicke S, Zeeman SC (2012). The simultaneous abolition of three starch hydrolases blocks transient starch breakdown in Arabidopsis. J Biol Chem.

[55] Yano R, Nakamura M, Yoneyama T, Nishida I (2005). Starch-related alpha-glucan/water dikinase is involved in the cold-induced development of freezing tolerance in Arabidopsis. Plant Physiol.

[56] Sicher R (2011). Carbon partitioning and the impact of starch deficiency on the initial response of Arabidopsis to chilling temperatures. Plant Sci.

[57] Fincher GB (1989). Molecular and cellular biology associated with endosperm mobilization in germinating cereal grains. Annu Rev Plant Physiol Plant Mol Biol.

[58] Yu TS, Zeeman SC, Thorneycroft D, Fulton DC, Dunstan H, Lue WL (2005). alpha-Amylase is not required for breakdown of transitory starch in Arabidopsis leaves. J Biol Chem.

[59] Santelia D, Trost P, Sparla F (2015). New insights into redox control of starch degradation. Curr Opin Plant Biol.

[60] Lao NT, Schoneveld O, Mould RM, Hibberd JM, Gray JC, Kavanagh TA (1999). An Arabidopsis gene encoding a chloroplast-targeted beta-amylase. Plant J.

[61] Kaplan F, Guy CL (2005). RNA interference of Arabidopsis beta-amylase8 prevents maltose accumulation upon cold shock and increases sensitivity of PSII photochemical efficiency to freezing stress. Plant J.

[62] Monroe JD, Storm AR, Badley EM, Lehman MD, Platt SM, Saunders LK (2014). Beta-Amylase1 and beta-amylase3 are plastidic starch hydrolases in Arabidopsis that seem to be adapted for different thermal, pH, and stress conditions. Plant Physiol.

[63] Wienkoop S, Morgenthal K, Wolschin F, Scholz M, Selbig J, Weckwerth W (2008). Integration of metabolomic and proteomic phenotypes analysis of data covariance dissects starch and RFO metabolism from Low and high temperature compensation response in Arabidopsis thaliana. Mol Cell Proteomics.

[64] Thomashow MF (2010). Molecular basis of plant cold acclimation: insights gained from studying the CBF cold response pathway. Plant Physiol.

[65] Gilmour SJ, Zarka DG, Stockinger EJ, Salazar MP, Houghton JM, Thomashow MF (1998). Low temperature regulation of the Arabidopsis CBF family of AP2 transcriptional activators as an early step in cold-induced COR gene expression. Plant J.

[66] Liu Q, Kasuga M, Sakuma Y, Abe H, Miura S, Yamaguchi-Shinozaki K (1998). Two transcription factors, DREB1 and DREB2, with an EREBP/AP2 DNA binding domain separate two cellular signal transduction pathways in drought- and low-temperature-responsive gene expression, respectively, in Arabidopsis. Plant Cell.

[67] Gilmour SJ, Fowler SG, Thomashow MF (2004). Arabidopsis transcriptional activators CBF1, CBF2, and CBF3 have matching functional activities. Plant Mol Biol.

[68] Jaglo-Ottosen KR, Gilmour SJ, Zarka DK, Schabenberger O, Thomashow MF (1998). Arabidopsis CBF1 overexpression induces COR genes and enhances freezing tolerance. Science.

[69] Rekarte-Cowie I, Ebshish OS, Mohamed KS, Pearce RS (2008). Sucrose helps regulate cold acclimation of Arabidopsis thaliana. J Exp Bot.

[70] Lastdrager J, Hanson J, Smeekens S (2014). Sugar signals and the control of plant growth and development. J Exp Bot.

[71] Monfared MM, Simon MK, Meister RJ, Roig-Villanova I, Kooiker M, Colombo L (2011). Overlapping and antagonistic activities of BASIC PENTACYSTEINE genes affect a range of developmental processes in Arabidopsis. Plant J.

[72] Lee SH, Chung GC, Jang JY, Ahn SJ, Zwiazek JJ (2012). Overexpression of PIP2;5 aquaporin alleviates effects of Low root temperature on cell hydraulic conductivity and growth in Arabidopsis. Plant Physiol.

[73] Schulze WX, Schneider T, Starck S, Martinoia E, Trentmann O (2012). Cold acclimation induces changes in Arabidopsis tonoplast protein abundance and activity and alters phosphorylation of tonoplast monosaccharide transporters. Plant J.

[74] Rampitsch C, Bykova NV (2012). The beginnings of crop phosphoproteomics: exploring early warning systems of stress. Front Plant Sci.

[75] Doerfler H, Lyon D, Nägele T, Sun X, Fragner L, Hadacek F (2013). Granger causality in integrated GC-MS and LC-MS metabolomics data reveals the interface of primary and secondary metabolism. Metabolomics.

[76] Weckwerth W, Wenzel K, Fiehn O (2004). Process for the integrated extraction, identification and quantification of metabolites, proteins and RNA to reveal their co‐regulation in biochemical networks. Proteomics.

[77] Colby T, Röhrig H, Harzen A, Schmidt J (2011). Modified metal-oxide affinity enrichment combined with 2D-PAGE and analysis of phosphoproteomes. Methods Mol Biol.

[78] Furuhashi T, Nukarinen E, Ota S, Weckwerth W (2014). Boron nitride as desalting material in combination with phosphopeptide enrichment in shotgun proteomics. Anal Biochem.

[79] Bodenmiller B, Mueller LN, Mueller M, Domon B, Aebersold R (2007). Reproducible isolation of distinct, overlapping segments of the phosphoproteome. Nat Methods.

[80] Cox J, Mann M (2008). MaxQuant enables high peptide identification rates, individualized p.p.b.-range mass accuracies and proteome-wide protein quantification. Nat Biotechnol.

[81] Cox J, Neuhauser N, Michalski A, Scheltema RA, Olsen JV, Mann M (2011). Andromeda: a peptide search engine integrated into the MaxQuant environment. J Proteome Res.

[82] Olsen JV, Blagoev B, Gnad F, Macek B, Kumar C, Mortensen P (2006). Global, in vivo, and site-specific phosphorylation dynamics in signaling networks. Cell.

[83] R Core Team (2013). R: A language and environment for statistical computing.

[84] Sun X, Weckwerth W (2012). COVAIN: a toolbox for uni-and multivariate statistics, time-series and correlation network analysis and inverse estimation of the differential Jacobian from metabolomics covariance data. Metabolomics.

[85] von Mering C, Jensen LJ, Snel B, Hooper SD, Krupp M, Foglierini M (2005). STRING: known and predicted protein-protein associations, integrated and transferred across organisms. Nucleic Acids Res.

