# Supplementary figures and images for: Integrative molecular profiling indicates a central role of transitory starch breakdown in establishing a stable C/N homeostasis during cold acclimation in two natural accessions of Arabidopsis thaliana
Source: BMC Plant Biol. 2015 Dec 1;15:284. doi: 10.1186/s12870-015-0668-1 (PMC4667452; doi:10.1186/s12870-015-0668-1)

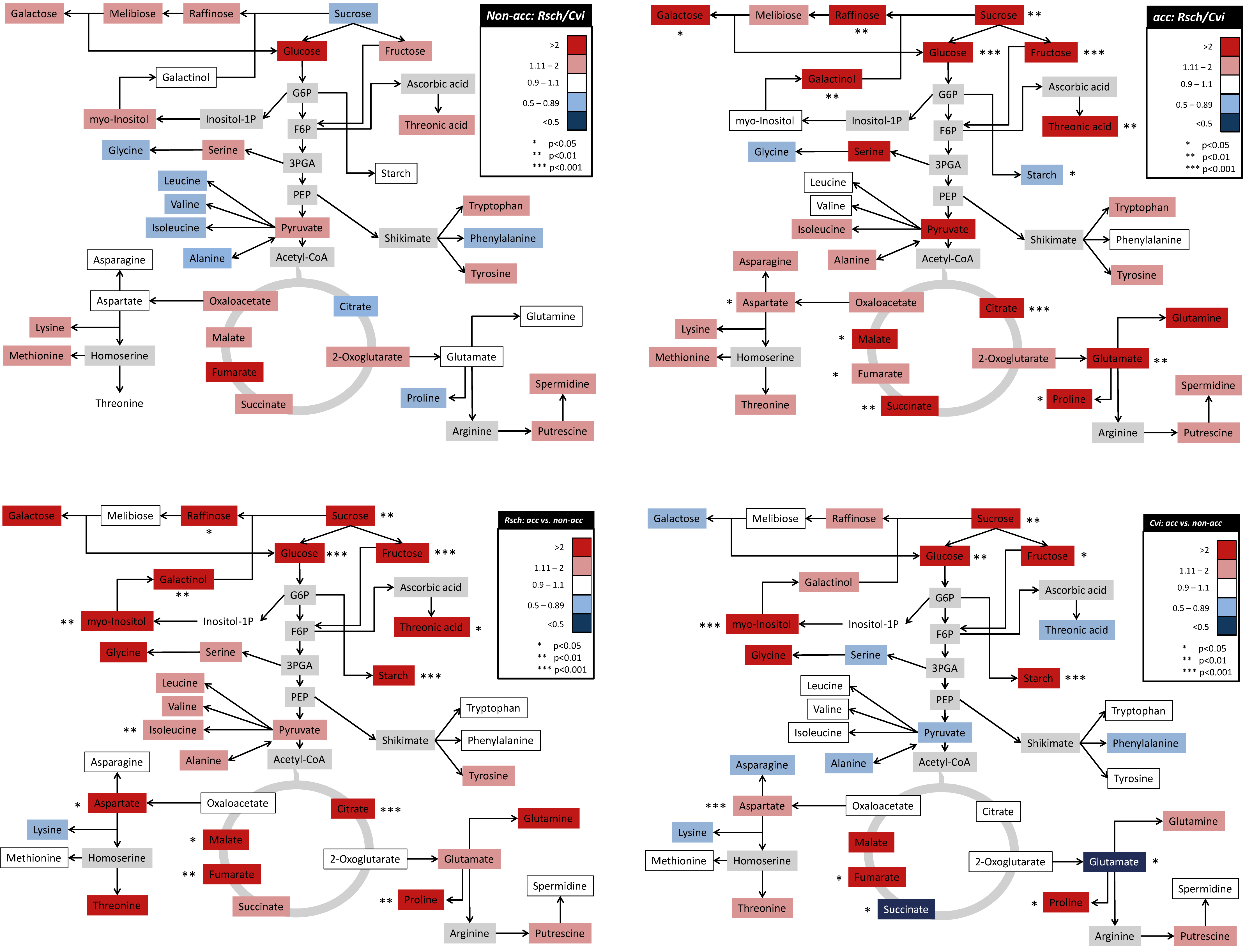

Supplement: Additional file 2: — Figure S1. Comparison of metabolite levels between non-acclimated and acclimated plants. Ratios were built by dividing the absolute mean values of metabolite levels of Rsch by levels of Cvi, or by dividing absolute mean values of metabolites of acc by na plants. Asterisks indicate significant differences as described in the figure. Grey-coloured metabolites were not experimentally analysed. (TIF 1649 kb) [file 12870_2015_668_MOESM2_ESM.tif]

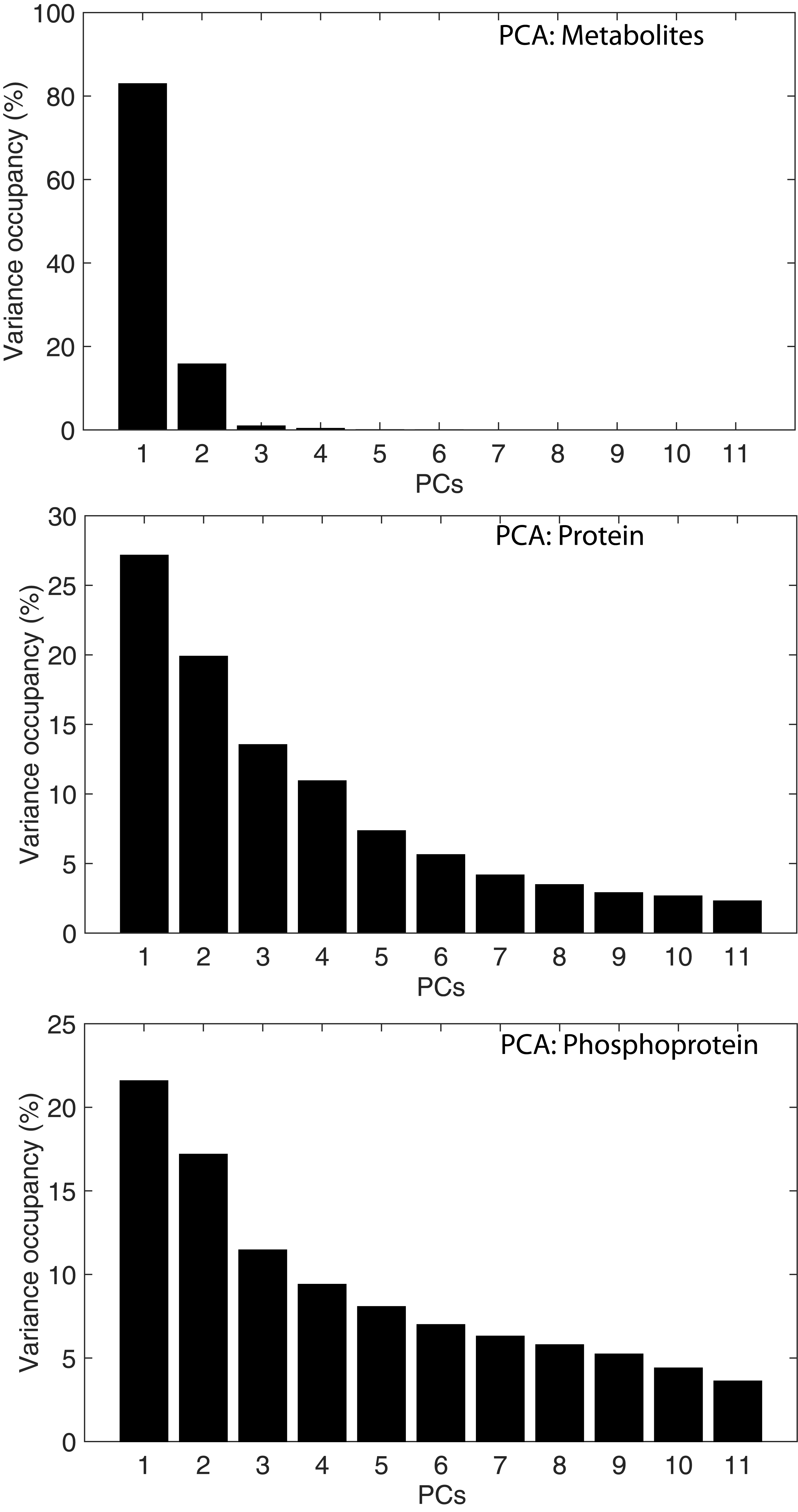

Supplement: Additional file 3: — Figure S2. Explained variances of principal components describing the separation by metabolites, protein and phosphoproteins in Fig. 2 of the main document. (TIF 692 kb) [file 12870_2015_668_MOESM3_ESM.tif]
